# Supplementary material for: Sex Disparity for Patients with Cutaneous Squamous Cell Carcinoma of the Head and Neck: A Systematic Review
Source: Cancers (Basel). 2022 Nov 26;14(23):5830. doi: 10.3390/cancers14235830 (PMC9740937; doi:10.3390/cancers14235830)
Supplement: Supplementary file 1 [file cancers-14-05830-s001.zip › Supplementary Table S1.pdf]

Supplementary Table S1: Summary of Included Articles

Author, Year	Country	Study period	Study design	Sample size- HNcSCC only	Sex (M, F) – HNcSCC only	Average age Mean (SD) (years)	Location of cancer (HNcSCC, Lip SCC, Ear SCC, Scalp SCC, Eyelid SCC)	Results (proportions of males to females or a comparison of incidence rates)
Alerić and Bauer, 2011	Croatia	1996-2010	Observational	158	M: 65; F: 93	Period 1: 73.2 (7.2). Period 2: 72.2 (7.6)[#]	HNcSCC	Higher proportion of females than males (41% vs 59% respectively)
								HNcSCC was 34.56% higher in females than males during study period 1 (1996-2000)
								HNcSCC was 8.74% higher in females than males during study period 2 (2006-2010)
Amir et al, 1992	Tanzania	1978-1988	Observational	337	M: 176; F: 158	Males: 50.2 Females: 49.3[#]	HNcSCC	Higher proportion of males than females (52% vs 48% respectively)
Baker et al, 2001	United Kingdom	1990-1995	Observational	183	M: 134; F: 47	78	HNcSCC	Higher proportion of males than females (73% vs 27% respectively)
Boi et al, 2003	Italy	1992-1997	Observational	524	M: 307, F: 217	74.8 (11.6)[#]	HNcSCC	Higher proportion of males than females (59% vs 41% respectively)
Brewster et al 2007	Scotland	1992-2003	Observational	3255	M: 2275; F: 980	Not reported	HNcSCC	Higher proportion of males than females (70% vs 30% respectively)
								Percentage of cSCC on the HN for males at each location: lip 2.7%, eyelid 1.9%, ear 22.2%, other parts of face 28.8, scalp and neck 17.2%
								Percentage of cSCC on the HN for females at each location: lip 2.4%, eyelid 2.2%, ear 1.1%, other parts of face 40%; scalp and neck 5.2%

								Anatomically, 73% of all cSCC were located on the HN for males. For females, 51% of all cSCC were found on the HN.
Buettner et al 1998	Australia	1996-1997	Observational	Not reported	Not reported	67 (median)#	HNcSCC	Anatomically, the head and neck was the most common site for cSCC for both males and females, making up 27.9% and 18.8% of total cSCC lesions respectively
								Incidence of cSCC on the scalp was 1216 per 100 000 body units for males and 118 per 100 000 per body units for females
								Incidence of cSCC on the ears, nose, cheek region was 16 321 per 100 000 body units for males and 7643 per 100 000 per body units for females
								Incidence of cSCC on the forehead, eyebrow, chin and jaw and preauricular region was 13 988 per 100 000 body units for males and 3608 per 100 000 per body units for females
								Incidence of cSCC on the lip, orbit, naso-labial region was 18 780 per 100 000 body units for males and 7308 per 100 000 per body units for females
								Incidence of cSCC on the neck was 4939 per 100 000 body units for males and 2686 per 100 000 per body units for females
Chiu et al, 2007	Taiwan (China)	1983-2003	Observational	66	M: 37; F: 29	70.4 (64.8)	Scalp cSCC	Higher proportion of males than females (56% vs 44% respectively)
Chuang et al, 1990	USA	1976-1984	Observational	138	M: 85; F: 53	71.4#	HNcSCC	Higher proportion of males than females (61.5% vs 39.5% respectively)
								Age standardised annual rates for 100 000 persons: scalp: males 6.1; females 0.0
								Age standardised annual rates for 100 000 persons: forehead: males 2.4; females 2.9

								Age standardised annual rates for 100 000 persons: nose/ eyelid chin: males 11.8; females 5.3
								Age standardised annual rates for 100 000 persons: temple: males 5.6; females 0.8
								Age standardised annual rates for 100 000 persons: cheek: males 9.2; females 5.8
								Age standardised annual rates for 100 000 persons: lip: males 2.7; females 1.5
								Age standardised annual rates for 100 000 persons: ear: males 11.8; females 0.6
								Age standardised annual rates for 100 000 persons: neck: males 3.7; females 0.0
Coebergh et al, 1991	The Netherlands	1975-1988	Observational	736	M: 512; F: 224	Males: lips 66; eyelids: 73, external ears: 74, face: 71 and neck and scalp: 73. Females: lips 67; eyelids: 72, external ears: 77, face: 76 and neck and scalp: 71.	HNcSCC	Higher proportion of males than females (70% vs 30% respectively)
								In males, anatomically, 18% of cSCC were found on the lips, 1% on the eyelids, 24% on the external ear, 34% on the face and 7% on the scalp/neck.
								In females, anatomically, 4% of cSCC were found on the lips, 4% on the eyelids, 5% on the external ear, 51% on the face and 7% on the scalp/neck.

Czarnecki et al, 1992	Australia	1989-1990	Observational	158	M: 119; F: 39	Melbourne males: 71.8; females: 72.2 Cairns males: 60.7; females: 57.6.	HNcSCC	Higher proportion of males than females (75% vs 25% respectively)<br>Males had more SCC on head and neck (compared with other anatomical regions) than females, but only statistically significant in Melbourne (not Cairns) (p<0.01).<br>The difference in the sex ratio between Melbourne and Cairns was statistically significant (p<0.02)
Dal et al, 2008	Sweden	1960-2004	Observational	Not reported	Not reported	Not reported	HNcSCC	Higher incidence of cSCC on all areas of the head and neck for males than females<br>Incidence of cSCC on the face/eyelids: ~11 per 100 000 in males and ~7 per 100 000 in females (in 2004)<br>Incidence of cSCC on the ears: ~5 per 100 000 in males and ~0.1 per 100 000 in females (in 2004)<br>Incidence of cSCC on the scalp and neck: ~4.2 per 100 000 in males and ~0.8 per 100 000 in females (in 2004)
Derebaşınlıoğlu 2022	Turkey	2000-2021	Observational	71	M: 53; F: 18	71.34 (11)[#]	HNcSCC	Higher proportion of males than females (75% vs 25% respectively)
Donaldson et al 2002	Australia	1992-2001	Observational	50	M: 33; F: 17	65	Eyelid cSCC	Higher proportion of males than females (66% vs<br>34% respectively)<br>Mean age of females was younger than males (63 years vs 66.8 years respectively).
Durmus Kocaaslan et al1 2019	Turkey	2010-2015	Observational	122	M: 86; F: 36	66.5 (19.76) Range: 9-95	HNcSCC	Higher proportion of males than females (70.5% vs 29.5% respectively)

Estall et al, 2017	Australia	1998-2010	Observational	235	M: 207; F: 28	79	Scalp HNcSCC	Higher proportion of males than females (88% vs 12% respectively)
Farah et al, 2022	USA	2010-2014	Observational	115	M: 105; F: 10	70 (median) Range:31-88	HNcSCC	Higher proportion of males than females (91% vs 9% respectively)
Faustina et al, 2004	USA	1952-2002	Observational	111	M: 89; F: 22	Males: 72.3 (median); Females: 75.6 (median) Range: 10-101	Eyelid cSCC	Higher proportion of males than females (80% vs 20% respectively)
Fears et al, 1982	USA	1971-1978	Observational	832	M: 631; F: 201	Not reported	HNcSCC	Higher proportion of males than females (76% vs 24% respectively)
								There was a small, non-significant increase in the rate of HNcSCC in males over the time period (4%)
								The increases in HNcSCC for females were higher in females than males, but again not significant (13%)
Franceschi et al, 1996	Italy	1976-1992	Observational	2350	M: 1352; F: 998	Not reported	HNcSCC	Higher proportion of males than females (57% vs 43% respectively)
								ASR of cSCC on the face for males was 11.6 per 10^5. ASR of cSCC on the face for females was 8.1 per 10^5.

								ASR of cSCC on the neck, ears and scalp for males was 6.3 per 10^5. ASR of cSCC on the neck, ears and scalp for females was 1.0 per 10^5.
Gallagher et al, 1990	Canada	1973-1987	Observational	1653	M: 1103; F: 550	Not reported	HNcSCC	Higher proportion of males than females (67% vs 33% respectively)
								The rate of male HNcSCC was higher than females. The rate also rose at a higher rate from 1973-1987 (Males: ~11 to 21 per 100 000; females ~6 to 8 per 100 000).
Givi et al, 2011	USA	1993-2007	Observational	51	M: 47; F: 4	79 (median) Range: 43-90	HNcSCC	Higher proportion of males than females (92% vs 8% respectively)
González et al, 2021	Argentina	2000-2018	Observational	163	M: 152; F: 11	76 Range: 43-96	Ear cSCC	Higher proportion of males than females (93% vs 7% respectively)
Gray et al , 1997	USA	1984-1992	Observational	397	M: 229; F: 168	64 (median); Range: 31-91	HNcSCC	Higher proportion of males than females (58% vs 42% respectively)

Harris et al, 2017	USA	1998-2014	Observational	212	M: 185, F: 27	70.4; Range: 32-94	HNcSCC	Higher proportion of males than females (87% vs 13% respectively)
								A cox univariate regression showed sex was not a predictor of recurrence (Female HR 0.97, p= 0.89)
								A cox multivariate regression showed sex was not a predictor of recurrence (Female HR 1.34, p= 0.64)
								A multinomial logistic regression demonstrated sex was not associated with nodal disease (Female HR 0.38, p= 0.27)
Hayes et al, 2007	Canada	1992-2001	Observational	2008	M: 1372, F: 716	Males: 72 (median) Females: 76 (median)[#]	HNcSCC	Higher proportion of males than females (68% vs 32% respectively)
								By anatomic location: Lips: M 182 vs F 32; Eyelid: M 50 vs F 35; Ear: M 382 vs F 31; Face M 616 vs F 566; Scalp/neck: M 142 vs F 52
								Among men, invasive SCC on the ear was the second most commonly diagnosed tumor (20.6%)
								Among women, invasive SCC was relatively uncommon on the ear (2.6% of tumors)
Hillström et al, 1970	Sweden	1958-1967	Observational	3816	M: 2399; F: 1420	Not reported	HNcSCC	Higher proportion of males than females (63% vs 37% respectively)
								Strikingly high rate of cancer on the ears for males compared to females. The scalp and neck are also more frequently affected in males.
Hollestein et al, 2012	The Netherlands	1989-2008	Observational	48 457	M: 31 846; F: 16 611	Not reported	HNcSCC	Higher proportion of males than females (58% vs 42% respectively)

								cSCC on the ear, neck and scalp were much more frequent in males than females
Iversen et al, 1999	Norway	1966-1995	Observational	7919	M: 4742; F: 3177	Males: 75 (median) Females: 77 (median)#	HNcSCC	Higher proportion of females than males (60% vs 40% respectively)
								In males, cancer of the auricle was the second most common site of tumours. This site was low and constant in females. (25% of invasive cancers in men, only 3% in women)
								For males with carcinoma of the auricle, the 5-year relative survival rate was higher than 80% for the whole period. However, among the females, the survival rate was low.
Jung SK et al, 2020	South Korea	1999-2016	Observational	602	M: 243; F: 359	Not reported	Eyelid cSCC	Higher proportion of females than males (60% vs 40% respectively)
Jung GW et al, 2010	Canada	1988-2007	Observational	13645	M: 9143; F: 4502	Not reported	HNcSCC	Higher proportion of males than females (67% vs 33% respectively)
								Anatomically, 74% of all cSCC were located on the HN for males. For females, 59% of all cSCC were found on the HN.
Kadakia et al, 2016	USA	1998-2013	Observational	93	M: 58; F: 35	64.8 Range: 27-98	Temporal region cSCC	Higher proportion of males than females (62% vs 38% respectively)
								Sex was not found to be a significant factor in parotid involvement (13 M vs 10 F, p=0.51)
Kampel et al, 2021	Israel	2008-2018	Observational	74	M: 56; F: 18	76 (10) Range: 45-93	HNcSCC	Higher proportion of males than females (76% vs 24% respectively)
								Males overall survival: HR 0.59 (95% CI 0.3-1.2), p=0.143
								Males disease-free survival: HR 0.8 (95% CI 0.3-2.5), p=0.697

Karagas et al, 1999	USA	1979-1994	Observational	628	M: 460, F: 168	Not reported	HNcSCC	Higher proportion of males than females (73% vs 27% respectively)
Karjalainen et al, 1989	Finland	1967-1981	Observational	2217	M: 1121; F: 1096	Males: 67.9-70.3 Females: 66.5-73.1#	HNcSCC	Higher proportion of males than females (55% vs 45% respectively)
								There were some differences in the subsite distribution of cSCC between males and females: the proportion of tumours of the ear, and scalp and neck was much higher in males (21.4% vs. 4.5% and 9.1% vs. 4.4%, respectively) and that of tumours of the face much higher in females (63.2% vs. 41.5%)
Karlin et al, 2020	USA	1998-2015	Observational	1252	M: 978, F: 274	Not reported	HNcSCC	Higher proportion of males than females (78% vs 22% respectively)
								Anatomically, males (compared with females) have a cSCC significantly more frequently located on scalp (excluding temple and forehead) (10.6% vs 3.3%, p<0.01); temple (7.2% vs 3.6%, p<0.05) and ear (29.2% vs 3.6%, p<0.01)
								Anatomically, females (compared with males) have a cSCC more frequently located on nose (25.2% vs 8.1%, p<0.01); cheek (29.2% vs 18.2%, p<0.01) and chin (7.1.2% vs 2.1%, p<0.05)
Kato et al, 2019	Japan	2004-2015	Observational	106	M: 50; F: 56	79.9 Range:42-104	HNcSCC	Higher proportion of females than males (53% vs 47% respectively)
Khalid et al, 2021	Australia	2012-2017	Observational	7589	M: 5436; F: 2153	76 (median) Range: 17-103#	HNcSCC	Higher proportion of males than females (72% vs 28% respectively)

Kim et al, 2020	USA	2000-2016	Observational	618	M: 366; F: 252	Not reported	HNcSCC	Higher proportion of males than females (59% vs 41% respectively)
								Anatomically, 51.7% of cSCCs developed on the head and neck in males, whereas this figure was 20.9% of all cSCC for females.
Košec et al, 2013	Croatia	1983-2007	Observational	103	M: 47; F: 56	74.5 Range: 47-92	HNcSCC	Higher proportion of females than males (54% vs 46% respectively)
								The average age of primary lesion occurrence was significantly greater in women (p<0.0012)
Kyrgidis et al, 2010	Greece	1996-2006	Observational	312	M: 145; F: 167	71.9 years Range: 26-95	HNcSCC	Higher proportion of females than males (54% vs 46% respectively)
Laprise et al, 2019	USA	1987-2014	Observational	167	M: 131; F: 36	Not reported	Lip cSCC	Higher proportion of males than females (65% vs 35% respectively)
Leibovitch et al, 2005	Australia	1993-2002	Observational	96	M: 62; F: 34	58 (15)	Lip cSCC	Higher proportion of males than females (65% vs 35% respectively, p<0.00001)
Levi et al, 1998	Switzerland	1976-1985	Observational	892	M: 553; F: 339	Not reported	HNcSCC	Higher proportion of males than females (63% vs 38% respectively)
								Anatomically, 81% of cSCCs developed on the head and neck in males, whereas this figure was 69% of all cSCC for females.
								Age-standardized rate of HNcSCC for males 13.0 per 100 000; For females: 5.3 per 100 000.
Lin et al, 2006	Taiwan (?China)	1979-1999	Observational	141	Not reported	61.8 Range: 0-96[#]	Eyelid cSCC	No association; male: female ratio=1
Luna-Ortiz et al, 2004	Mexico	1990-2000	Observational	82	M: 57; F: 25	70 Range: 14-106[#]	Lip cSCC	Higher proportion of males than females (70% vs 30% respectively)
Marks et al, 1993	Australia	1990	Observational	67	M: 54; F: 13	Not reported	HNcSCC	Higher proportion of males than females (80.5% vs 19.5% respectively)

								In males, 48% of all cSCC were located on the HN. In females, 24% of all cSCC were located on the HN.
McDowell et al, 2016	Australia	2000-2014	Observational	132	M: 121; F: 11	76 (median) Range: 27-98	HNcSCC	Higher proportion of males than females (92% vs 8% respectively)
Mehta et al, 2021	USA	1795-2016	Observational	15 171	M: 12 259; F: 2912	Not reported	Lip cSCC	Higher proportion of males than females (81% vs 19% respectively)
								The proportion of lip sCC in females increased during the reporting period from 8.4% in 1975-79 to 26.1% in 2015-16.
								The proportion of lip sCC in males decreased during the reporting period from 91.6% in 1975-79 to 73.9% in 2015-16, due to decreasing rates of cSCC in males during this time period.
Mistry et al, 2012	Canada	1993-2005	Observational	99	M: 66; F: 33	1972: 76.2 2001: 64.1#	HNcSCC	Higher proportion of males than females (67% vs 33% respectively)
Mooney et al, 2021	Australia	1989-2017	Observational	200	M: 182; F: 18	Not reported	HNcSCC	Higher proportion of males than females (91% vs 9% respectively)
								Males were 0.58 times less likely to have disease free survival than females HR 0.58, 95% CI 0.34-0.99
								No difference in overall survival between males and females
								No difference in disease free survival between males and females when a multivariable analysis was conducted.
Mora et al, 1981	USA	1948-1979	Observational	77	M: 29; F: 48	Males: 60.1	HNcSCC	Higher proportion of females than males (62% vs 38% respectively)

						Range: 15-93<br>Females: 57.6<br>Range: 11-100[#]		
Moser et al, 2020	Austria	2005-2015	Observational	98	M: 80: F: 18	Males: 74.6 (10.6)<br>Females 81.2 (7.3)	Ear cSCC	Significantly higher proportion of males than females (81% vs 19% respectively, p=0.001)<br>Females with cSCC of the pinna were significantly older than males (females had a median age of 81.2 ± 7.3 years whereas males were 74.6 ± 10.6 years old)<br>In females, the most common tumour site of cSCC was the helix (66.7%)<br>In males, the helix (50%) was the most frequent location of cSCC.
Mourouzis et al, 2009	United Kingdom	200-2002	Observational	194	M: 143; F: 51	Range: 62-104	HNcSCC	Higher proportion of males than females (74% vs 26% respectively)<br>The site distribution of the cSCCs has a gender disparity- in males, more than half (54%) of cSCCs were excised from the scalp and ear, whereas in females, most (60%) occurred in the forehead and cheek
Muzic et al, 2017	USA	2000-2010	Observational	Not reported	Not reported	70.5 (13.4)[#]	HNcSCC	Approximately 50% of all cSCC are found on the head and neck for females, but this figure is ~68% for males
Nguyen et al, 2014	USA	1976-2009	Observational	1586	Not reported	NHS I 65.5 (8.4); NHS II 48.3 (6.32); HPFS: 67.6 (8.8)[#]	HNcSCC	HNcSCC lesions accounted for a larger proportion of all invasive lesions for males than for females (60% HPFS vs 43% NHS, p<0.001; 60%HPFS vs 48% NHS II, p<0.001)

Norval et al, 2014	South Africa	2000-2004	Observational	Not reported	Not reported	Not reported	HNcSCC	Annual incidence of HNcSCC in males was 20.8 per 100 000, whereas in females it's 8.5 per 100 000.
Omari et al, 2006	Jordan	1997-2001	Observational	52	M: 38; F: 14	Females: 49 (median) Males (median): 70#	HNcSCC	Higher proportion of males than females (73% vs 27% respectively)<br>In males, 72% of all cSCC were located on the HN. In females, 64% of all cSCC were located on the HN.
Osterlind et al, 1988	Denmark	1978-1982	Case- control	1387	M: 973; F: 414	Not reported	HNcSCC	Higher proportion of males than females (70% vs 30% respectively)<br>Incidence rate for males: 4.7 per 100 000. Incidence rate for females: 1.5 per 100 000.<br>For males: face/scalp and neck make up 76.3% of all SCC in terms of location. For females: face/scalp and neck make up 66.7% of all SCC in terms of location.
Papadopoulos O et al, 2007	Greece	1983-2005	Observational	467	M: 384; F: 83	Not reported	Lip cSCC	Higher proportion of males than females (82% vs 18% respectively)
Papadopoulos O et al, 2009	Greece	1985-2006	Observational	109	Not reported	65.9 Range: 13-97	Nasal cSCC	There was no significant difference between males and females regarding the location of the cSCC on the nose.
Pyne et al, 2018	Australia	2009-2015	Observational	691	Not reported	71 (median)#	HNcSCC	There was a difference between men and women regarding poorly differentiated SCCs. For men, they were most frequently on the scalp (21%), cheek/chin (20%), forehead (13%) and ear (9%). For women, poorly differentiated SCC was most frequent on the cheek/chin (19%).
Quigley et al, 2019	Ireland	2005-2015	Observational	528	M: 290, F: 238	Not reported	Eyelid cSCC	Higher proportion of males than females (55% vs 45% respectively)<br>ASR for males: 2.10 per 100 000; ASR for females 1.39 per 100 000.

								ASR increased significantly for women at a rate of 0.07 cases per 100 000 annually (p<0.0005)
								ASR increased for men, however not statistically significant (p=0.18)
Robsahm et al, 2015	Norway	1963-2011	Observational	Not reported	Not reported	Not reported	HNcSCC	The incidence of cSCC of the head/face in males is 10.4 per xx and 7.3 per xx for females.
								The incidence of cSCC of the ear in males is 3.9 per xx and 0.2 per xx for females.
								The annual percentage change for head/face cSCC increased at a faster rate in females than males (6.1% vs 5.1% respectively)
Seretis et al, 2010	Greece	2004-206	Observational	54	M: 38, F: 16	70.3 (11.8)[#]	HNcSCC	Higher proportion of males than females (70% vs 30% respectively)
								The difference between men and women regarding age of SCC diagnosis and subsequent treatment was statistically signficant (t= -3.015, p=0.004)
Silapunt et al, 2005	USA	1997-2002	Observational	117	M: 112; F: 5	71 Range: 34-90	Ear cSCC	Higher proportion of males than females (96% vs 4% respectively)
Singer et al, 2017	Germany	2003-2012	Observational	4869	M: 3049; F: 1820	Males: 71.8 Females: 72.7[#]	Lip cSCC	Higher proportion of males than females (63% vs 37% respectively)
								For males, cSCC of the lip made up 55.6% of all NMSC, whereas this was only 26.5% for females.
Soysal et al, 2006	Turkey	1997-2006	Observational	76	M: 41; F: 35	64.26 (3.54); Range: 11-93	Eyelid cSCC	Higher proportion of males than females (54% vs 46% respectively)
Staples et al, 2006	Australia	2002	Observational	172	M: 120; F: 52	Not reported	HNcSCC	Higher proportion of males than females (70% vs 30% respectively)

								The RTD for HNcSCC men: 5.55 per 100 000; women: 3.72 per 100 000
Subramaniam et al, 2017	Australia	2010-2013	Observational	435	Not reported	Not reported	HNcSCC	The relative tumour density of cSCC was 10 times higher on the ears for males than females and 5 times higher on the scalp
Sweeny et al, 2014	USA	2001-2012	Observational	218	M: 192; F: 26	71<br>Range: 35-95	HNcSCC	Higher proportion of males than females (88% vs 12% respectively)
Szcwczyk et al, 2015	Poland	Not reported	Observational	100	M: 66; F: 34	74.6 (12)	HNcSCC	Higher proportion of males than females (66% vs 34% respectively)
								In males, the most common localisation was the auricle (25 of 66 cases, 38%) and the lip (16 cases. 24%)
								In females, the most common localisation was the nose (8 of 34 cases, 23%) and the buccal region (8 cases. 23%)
Thomas et al, 2017	Australia	2007	Observational	110	M: 69; F: 41	63.4[#]	HNcSCC	Significantly higher proportion of males than females (63% vs 37% respectively, p=0.005)
Tseng et al, 2017	Taiwan (China)	1991-2013	Observational	112	M: 99; F: 13	59.41 (12.6)#	Lip cSCC	Higher proportion of males than females (88% vs 12% respectively)
								Disease-specific survival Female: 100%; Males: 85.8%
								Disease-free survival Female 56.8%; Males: 73.5% (Not significant, p=0.45)
van der Leest et al, 2018	The Netherlands	1989-2009	Observational	48 247	M: 31 393;<br>F: 16 854	Not reported	HNcSCC	Higher proportion of males than females (65% vs 35% respectively)
Veness et al, 2006	Australia	1980-2005	Observational	266	M: 219; F: 47	69 (median)<br>Range: 34-95	HNcSCC	Higher proportion of males than females (82% vs 18% respectively)
Warnig et al, 2021	Germany	2006-2016	Observational	1194	M: 804; F: 390	80.8 (8.3)	HNcSCC	Higher proportion of males than females (67% vs 33% respectively)

								HNcSCC were most commonly located on the ears (23.6%) and scalp (22.6%) for males
								HNcSCC were most commonly located on the cheek (26.7%) and forehead (20%) for males. Only 4.8% of HNcSCC were found on the ears in females
								Females were older than males at diagnosis
Wassberg et al, 2001	Sweden	1961-1995	Observational	Not reported	Not reported	Not reported	HNcSCC	Males had a higher incidence rate at all time points in the study period.
								The incidence rate of HNcSCC in males increased from 3.1 per 100 000 in 1961 to 14.6 per 100 000 in 1995 (+370%)
								The incidence rate of HNcSCC in females increased from 2.3 per 100 000 in 1961 to 5.0 per 100 000 in 1995 (+119%)
Wawrzynski et al, 2018	England (?UK)	2000-2014	Observational	4022	Not reported	Not reported	Eyelid cSCC	The relative risk of being diagnosed with an eyelid cSCC is 1.9 (95% CI 1.5, 2.3) times greater in males than females, with the risk remaining consistent over the time period studied.
Wiser et al, 2016	Israel	2009-2011	Observational	621	M: 408; F: 213	75.2 (11.2)	HNcSCC	Higher proportion of males than females (66% vs 34% respectively)
								Females were 2.6 years older than males at diagnosis (p=0.005).
								Significant differences were found in the anatomic location of HNcSCC by gender (p<0.001): cSCC on the scalp and ear were observed more in males than females (22.1% vs 6.1% and 20.3% vs 3.3% respectively, p<0.001)

								Incidence rates: M: 106.2 per 1 000 000; F: 54.3 per 1 000 000 (Approximately 2:1 male to female incidence ratio)
Yakubu et al, 1995	Nigeria	1978-1989	Observational	163	M: 98; F: 65	40.6 Range: 5 months – 75 years#	HNcSCC	Higher proportion of males than females (60% vs 40% respectively)
Youl et al, 2011	Australia	2005	Observational	677	M: 424; F: 253	Not reported	HNcSCC	Higher proportion of males than females (63% vs 37% respectively)
								Males had significantly more SCCs excised from the scalp and ears ($p<0.001$)
Zanetti et al, 1996	Italy	1989-1993	Observational	172	M: 142; F: 30	Not reported	HNcSCC	Higher proportion of males than females (83% vs 17% respectively)

#not specifically HNcSCC mean/median. ASR= Age Standardised Rate; HNcSCC= Head and neck cutaneous squamous cell carcinoma; HN= Head and Neck; RTD= Relative tumour density. #Mean not specific for HNcSCC; NHS= Nurse's Health Study; HPFS= Health Professionals Follow-Up Study
