# Supplementary material for: Sex Disparity for Patients with Cutaneous Squamous Cell Carcinoma of the Head and Neck: A Systematic Review
Source: Cancers (Basel). 2022 Nov 26;14(23):5830. doi: 10.3390/cancers14235830 (PMC9740937; doi:10.3390/cancers14235830)
Supplement: Supplementary file 1 [file cancers-14-05830-s001.zip › Supplementary Table S2.pdf]

Supplementary Table S2. Risk of Bias

Author, Year	1. Was the research question or objective in this paper clearly stated?	2. Was the study population clearly specified and defined?	3. Was the participation rate of eligible persons at least 50%?	4. Were all the subjects selected or recruited from the same or similar populations (including the same time period)? Were inclusion and exclusion criteria for being in the study pre-specified and applied uniformly to all participants?	5. Was a sample size justification, power description, or variance and effect estimates provided?	6. For the analyses in this paper, were the exposure(s) of interest measured prior to the outcome(s) being measured?	7. Was the time frame sufficient so that one could reasonably expect to see an association between exposure and outcome if it existed?	8. For exposures that can vary in amount or level, did the study examine different levels of the exposure as related to the outcome (eg, categories of exposure, or exposure measured as continuous variable)?	9. Were the exposure measures (independent variables) clearly defined, valid, reliable, and implemented consistently across all study participants?	10. Was the exposure(s) assessed more than once over time?	11. Were the outcome measures (dependent variables) clearly defined, valid, reliable, and implemented consistently across all study participants?	12. Were the outcome assessors blinded to the exposure status of participants?	13. Was loss to follow-up after baseline 20% or less?	14. Were key potential confounding variables measured and adjusted statistically for their impact on the relationship between exposure(s) and outcome(s)?	Total Scores	Quality Rating (Poor, Fair, Good)
Alerić and Bauer, 2011	Yes	Yes	NR	Yes	NR	NA	Yes	NA	NA	NA	Yes	NR	Yes	Yes	7	Fair
Amir et al, 1992	Yes	Yes	Yes	Yes	NR	NA	Yes	NA	NA	NA	Yes	NR	Yes	Yes	8	Fair

Baker et al, 2001	Yes	Yes	NR	Yes	NR	NA	Yes	NA	NA	NA	Yes	NR	Yes	Yes	7	Fair
Boi et al, 2003	Yes	Yes	NR	Yes	NR	NA	Yes	NA	NA	NA	Yes	NR	Yes	Yes	7	Fair
Brewster et al 2007	Yes	Yes	NR	Yes	NR	NA	Yes	NA	NA	NA	Yes	NR	Yes	Yes	7	Fair
Buettner et al 1998	Yes	Yes	NR	Yes	NR	NA	Yes	NA	NA	NA	Yes	NR	Yes	NR	6	Fair
Chiu et al, 2007	Yes	Yes	NR	Yes	NR	NA	Yes	NA	NA	NA	Yes	NR	Yes	NR	7	Fair
Chuang et al, 1990	Yes	Yes	NR	Yes	NR	NA	Yes	NA	NA	NA	Yes	NR	Yes	Yes	7	Fair
Coebergh et al, 1991	Yes	Yes	NR	Yes	NR	NA	Yes	NA	NA	NA	Yes	NR	Yes	Yes	7	Fair
Czarnecki et al, 1992	Yes	Yes	Yes	Yes	NR	NA	Yes	NA	NA	NA	Yes	NR	Yes	NR	7	Fair
Dal et al, 2008	Yes	Yes	NR	Yes	NR	NA	Yes	NA	NA	NA	Yes	NR	Yes	Yes	7	Fair
Derebaşınlıoğlu 2022	Yes	Yes	NR	Yes	NR	NA	Yes	NA	NA	NA	Yes	NR	Yes	Yes	7	Fair
Donaldson et al 2002	Yes	Yes	NR	Yes	NR	NA	Yes	NA	NA	NA	Yes	NR	Yes	NR	6	Fair
Durmus Kocaaslan et al 2019	Yes	Yes	NR	Yes	Yes	NA	Yes	NA	NA	NA	Yes	NR	Yes	Yes	8	Fair
Estall et al, 2017	Yes	Yes	NR	Yes	NR	NA	Yes	NA	NA	NA	Yes	NR	Yes	Yes	7	Fair
Farah et al, 2022	Yes	Yes	NR	Yes	NR	NA	Yes	NA	NA	NA	Yes	NR	Yes	Yes	7	Fair
Faustina et al, 2004	Yes	Yes	NR	Yes	NR	NA	Yes	NA	NA	NA	Yes	NR	Yes	Yes	7	Fair

Fears et al, 1982	Yes	Yes	NR	Yes	NR	NA	Yes	NA	NA	NA	Yes	NR	Yes	Yes	7	Fair
Franceschi et al, 1996	Yes	Yes	NR	Yes	Yes	NA	Yes	NA	NA	NA	Yes	NR	Yes	NR	7	Fair
Gallagher et al, 1990	Yes	Yes	NR	Yes	NR	NA	Yes	NA	NA	NA	Yes	NR	Yes	NR	5	Fair
Givi et al, 2011	Yes	Yes	NR	Yes	NR	NA	Yes	NA	NA	NA	Yes	NR	Yes	Yes	7	Fair
González et al, 2021	Yes	Yes	Yes	Yes	NR	NA	Yes	NA	NA	NA	Yes	NR	Yes	Yes	8	Fair
Gray et al , 1997	Yes	Yes	NR	Yes	NR	NA	Yes	NA	NA	NA	Yes	NR	Yes	NR	6	Fair
Harris et al, 2017	Yes	Yes	NR	Yes	Yes	NA	Yes	NA	NA	NA	Yes	NR	Yes	Yes	8	Fair
Hayes et al, 2007	Yes	Yes	NR	Yes	NR	NA	Yes	NA	NA	NA	Yes	NR	Yes	Yes	7	Fair
Hillström et al, 1970	Yes	Yes	No	Yes	NR	NA	Yes	NA	NA	NA	Yes	NR	Yes	Yes	6.5	Fair
Hollestein et al, 2012	Yes	Yes	NR	Yes	NR	NA	Yes	NA	NA	NA	Yes	NR	Yes	Yes	7	Fair
Iversen et al, 1999	Yes	Yes	Yes	Yes	NR	NA	Yes	NA	NA	NA	Yes	NR	Yes	Yes	7	Fair
Jung SK et al, 2020	Yes	Yes	Yes	Yes	Yes	NA	Yes	NA	NA	NA	Yes	NR	Yes	NR	8	Fair
Jung et al, 2010	Yes	Yes	CD	Yes	NR	NA	Yes	NA	NA	NA	Yes	NR	Yes	Yes	7	Fair
Kadakia et al, 2016	Yes	Yes	NR	Yes	NR	NA	Yes	NA	NA	NA	Yes	NR	Yes	Yes	7	Fair
Kampel et al, 2021	Yes	Yes	NR	Yes	NR	NA	Yes	NA	NA	NA	Yes	NR	Yes	Yes	7	Fair

Karagas et al, 1999	Yes	Yes	NR	Yes	Yes	NA	Yes	NA	NA	NA	Yes	NR	Yes	Yes	8	Fair
Karjalainen et al, 1989	Yes	Yes	Yes	Yes	Yes	NA	Yes	NA	NA	NA	Yes	NR	Yes	Yes	9	Fair
Karlin et al, 2020	Yes	Yes	No	Yes	NR	NA	Yes	NA	NA	NA	Yes	NR	Yes	Yes	7.5	Fair
Kato et al, 2019	Yes	Yes	Yes	Yes	NR	NA	Yes	NA	NA	NA	Yes	NR	Yes	Yes	8	Fair
Khalid et al, 2021	Yes	Yes	No	Yes	NR	NA	Yes	NA	NA	NA	Yes	NR	Yes	Yes	7.5	Fair
Kim et al, 2020	Yes	Yes	NR	Yes	NR	NA	Yes	NA	NA	NA	Yes	NR	Yes	Yes	7	Fair
Košec et al, 2013	Yes	Yes	Yes	Yes	NR	NA	Yes	NA	NA	NA	Yes	NR	Yes	Yes	8	Fair
Kyrgidis et al, 2010	Yes	Yes	No	Yes	NR	NA	NA	NA	NA	NA	Yes	NR	Yes	Yes	7.5	Fair
Laprise et al, 2019	Yes	Yes	NR	Yes	No	NA	Yes	NA	NA	NA	Yes	NR	Yes	Yes	7.5	Fair
Leibovitch et al, 2005	Yes	Yes	NR	Yes	No	NA	Yes	NA	NA	NA	Yes	NR	Yes	Yes	7.5	Fair
Levi et al, 1998	Yes	Yes	NR	Yes	NR	NA	NA	NA	NA	NA	Yes	NR	Yes	NR	6	Fair
Lin et al, 2006	Yes	Yes	Yes	Yes	NR	NR	Yes	NA	NA	NA	Yes	NR	Yes	Yes	7	Fair
Luna-Ortiz et al, 2004	Yes	Yes	NR	Yes	NR	NA	Yes	NA	NA	NA	Yes	NR	Yes	Yes	7	Fair
Marks et al, 1993	Yes	Yes	NR	Yes	NR	NA	Yes	NA	NA	NA	Yes	NR	Yes	NR	6	Fair
McDowell et al, 2016	Yes	Yes	NR	Yes	NR	NA	NA	NA	NA	NA	Yes	NR	Yes	Yes	7	Fair
Mehta et al, 2021	Yes	Yes	Yes	Yes	NR	NA	Yes	NA	NA	NA	Yes	NR	Yes	Yes	8	Fair
Mistry et al, 2012	Yes	Yes	NR	Yes	NR	NA	Yes	NA	NA	NA	Yes	NR	Yes	Yes	7	Fair

Mooney et al, 2021	Yes	Yes	NR	Yes	No	NA	Yes	NA	NA	NA	Yes	NR	Yes	Yes	7.5	Fair
Mora et al, 1981	Yes	Yes	NR	Yes	NR	NA	Yes	NA	NA	NA	Yes	NR	Yes	NR	6	Fair
Moser et al, 2020	Yes	Yes	NR	Yes	NR	NA	Yes	NA	NA	NA	Yes	NR	Yes	Yes	7	Fair
Mourouzis et al, 2009	Yes	Yes	Yes	Yes	NR	NA	Yes	NA	NA	NA	Yes	NR	Yes	Yes	8	Fair
Muzic et al, 2017	Yes	Yes	NR	Yes	Yes	NA	Yes	NA	NA	NA	Yes	NR	Yes	Yes	8	Fair
Nguyen et al, 2014	Yes	Yes	NR	Yes	NR	NA	Yes	NA	NA	NA	Yes	NR	Yes	Yes	7	Fair
Norval et al, 2014	Yes	Yes	NR	Yes	NR	NA	Yes	NA	NA	NA	Yes	NR	Yes	Yes	7	Fair
Omari et al, 2006	Yes	Yes	NR	Yes	No	NA	Yes	NA	NA	NA	Yes	NR	Yes	NR	6.5	Fair
Osterlind et al, 1988	Yes	Yes	NR	Yes	NR	NA	Yes	NA	NA	NA	Yes	NR	Yes	NR	6	Fair
Papadopoulos et al, 2007	Yes	Yes	NR	Yes	NR	NA	Yes	NA	NA	NA	Yes	NR	Yes	NR	6	Fair
Papadopoulos et al, 2009	Yes	Yes	Yes	Yes	NA	NA	Yes	NA	NA	NA	Yes	NR	Yes	Yes	8	Fair
Pyne et al, 2018	Yes	Yes	NR	Yes	NR	NA	Yes	NA	NA	NA	Yes	NR	Yes	Yes	7	Fair
Quigley et al, 2019	Yes	Yes	NR	Yes	Yes	NA	Yes	NA	NA	NA	Yes	NR	Yes	Yes	8	Fair
Robsahm et al, 2015	Yes	Yes	NR	Yes	NR	NA	Yes	NA	NA	NA	Yes	NR	Yes	Yes	7	Fair
Seretis et al, 2010	Yes	Yes	NR	Yes	No	NA	Yes	NA	NA	NA	Yes	NR	Yes	Yes	7.5	Fair
Silapunt et al, 2005	Yes	Yes	CD	Yes	NR	NA	Yes	NA	NA	NA	Yes	NR	Yes	Yes	7	Fair

Singer et al, 2017	Yes	Yes	NR	Yes	NR	NA	Yes	NA	NA	NA	Yes	NR	Yes	Yes	7	Fair
Soysal et al, 2006	Yes	Yes	NR	Yes	NR	NA	Yes	NA	NA	NA	Yes	NR	Yes	NR	6	Fair
Staples et al, 2006	Yes	Yes	NR	Yes	NR	NA	Yes	NA	NA	NA	Yes	NR	Yes	NR	6	Fair
Subramaniam et al, 2017	Yes	Yes	NR	Yes	Yes	NA	Yes	NA	NA	NA	Yes	NR	Yes	Yes	8	Fair
Sweeny et al, 2014	Yes	Yes	NR	Yes	NR	NA	Yes	NA	NA	NA	Yes	NR	Yes	Yes	7	Fair
Szcwczyk et al, 2015	Yes	Yes	NR	Yes	No	NA	Yes	NA	NA	NA	Yes	NR	Yes	NR	7.5	Fair
Thomas et al, 2017	Yes	Yes	Yes	Yes	NR	NA	Yes	NA	NA	NA	Yes	NR	Yes	Yes	8	Fair
Tseng et al, 2017	Yes	Yes	NR	Yes	No	NA	Yes	NA	NA	NA	Yes	NR	Yes	Yes	7.5	Fair
van der Leest et al, 2018	Yes	Yes	NR	Yes	Yes	NA	Yes	NA	NA	NA	Yes	NR	Yes	Yes	8	Fair
Veness et al, 2006	Yes	Yes	NR	Yes	NR	NA	Yes	NA	NA	NA	Yes	NR	Yes	NR	6	Fair
Warnig et al, 2021	Yes	Yes	Yes	Yes	NR	NA	Yes	NA	NA	NA	Yes	NR	Yes	Yes	8	Fair
Wassberg et al, 2001	Yes	Yes	NR	Yes	NR	NA	Yes	NA	NA	NA	Yes	NR	Yes	Yes	7	Fair
Wawrzynski et al, 2018	Yes	Yes	Yes	Yes	NR	NA	Yes	NA	NA	NA	Yes	NR	Yes	Yes	8	Fair
Wiser et al, 2016	Yes	Yes	NR	Yes	NR	NA	Yes	NA	NA	NA	Yes	NR	Yes	Yes	7	Fair
Yakubu et al, 1995	Yes	Yes	NR	Yes	NR	NA	Yes	NA	NA	NA	Yes	NR	Yes	NR	6	Fair

Youl et al, 2011	Yes	Yes	NR	Yes	NR	NA	Yes	NA	NA	NA	Yes	NR	Yes	Yes	7	Fair
Zanetti et al, 1996	Yes	Yes	Yes	Yes	NR	Yes	Yes	NA	Yes	NA	Yes	Yes	Yes	Yes	11	Good
